# Differences in clinical characteristics of early-onset and late-onset severe acute respiratory syndrome coronavirus 2 infections in neonates

**DOI:** 10.1007/s00431-024-05433-6

**Published:** 2024-01-18

**Authors:** Yi-Xin Wu, Dan-Dan Wang, Ru-Qi Zhao, Ou-Xuan Jin, Jing-Yun Yang, Mei-Xian Zhang, Li-Zhen Wang

**Affiliations:** 1 Department of Pediatrics, Taizhou Hospital of Zhejiang Province affiliated to Wenzhou Medical University, 150 Ximen Street, Linhai, 317000 Zhejiang China; 2 Evidence-based Medicine Center, Taizhou Hospital of Zhejiang Province Affiliated to Wenzhou Medical University, 150 Ximen Street, Linhai, 317000 Zhejiang Province China

**Keywords:** COVID-19, Neonatal intensive care unit, Neonate, Pneumonia, SARS-CoV-2

## Abstract

Differences in clinical characteristics of early-onset and late-onset severe acute respiratory syndrome coronavirus 2 (SARS-CoV-2) infections in neonates remain unclear. This study aimed to determine whether there are differences in the main clinical, radiological, and laboratory features of early-onset and late-onset SARS-CoV-2 infections in neonates. This single-center, prospective cohort study enrolled neonates with SARS-CoV-2 infection from December 7, 2022, to January 3, 2023, and evaluated their clinical characteristics during hospitalization. All neonates (*N* = 58) infected with SARS-CoV-2 within 28 days of birth who were admitted to the neonatal intensive care unit of Taizhou Hospital were included. These neonates were classified into the early-onset (diagnosed within 7 days of birth) and late-onset (diagnosed more than 7 days after birth) groups. The symptoms, treatment, and prognosis of SARS-CoV-2 infection were the main study outcomes. The incidence of hospitalization attributable to SARS-CoV-2 infection was 10.6% (58 of 546 neonates) in Linhai. Sixteen (28%) of the 58 SARS-CoV-2 infections were early-onset cases, and 42 (72%) were late-onset cases. The common symptoms among the late-onset group were fever (*p* < 0.001) and cough (*p* < 0.001). Neonates with late-onset SARS-CoV-2 infection (*p* < 0.001) were significantly more likely to develop pneumonia.

*  Conclusion*: The clinical symptoms and rates of pneumonia caused by SARS-CoV-2 infection in neonates differed between the early-onset and late-onset groups. Different clinical management is necessary for neonates with early-onset and late-onset SARS-CoV-2 infections.

**Table:** 

**What is Known:** *• Neonates are susceptible to severe acute respiratory syndrome coronavirus 2 (SARS-CoV-2).* *• Differences in clinical characteristics of early-onset and late-onset SARS-CoV-2 infections in neonates remain unclear.*	
**What is New:** *• Fever and cough were the most common symptoms among neonates with late-onset infection.* *• Neonates with late-onset SARS-CoV-2 infection were more likely to develop pneumonia.*

**What is Known:**

*• Neonates are susceptible to severe acute respiratory syndrome coronavirus 2 (SARS-CoV-2).*

*• Differences in clinical characteristics of early-onset and late-onset SARS-CoV-2 infections in neonates remain unclear.*

**What is New:**

*• Fever and cough were the most common symptoms among neonates with late-onset infection.*

*• Neonates with late-onset SARS-CoV-2 infection were more likely to develop pneumonia.*

## Introduction

The coronavirus disease (COVID-19) pandemic comprised a global outbreak of an infectious disease caused by the severe acute respiratory syndrome coronavirus 2 (SARS-CoV-2) [1]. Since the reporting of the first case in December 2019 until May 2023, SARS-CoV-2 infections were diagnosed in more than 750 million people and resulted in the death of more than 6.5 million people worldwide [2]. All age groups, including newborns and infants, are susceptible to SARS-CoV-2 [3–5]. The severity of symptoms experienced by patients with SARS-CoV-2 infection varies with age, and previous studies have suggested that SARS-CoV-2 infection is less severe in children than in adults [6].

A systematic review showed that most neonates infected with SARS-CoV-2 within 28 days of birth presented with mild-to-moderate symptoms [7]. Newborns with early-onset SARS-CoV-2 infection are exposed to the virus in a different way than those with late-onset infection; those with early-onset infection can be infected with SARS-CoV-2 through close contact with others and vertically infected with the virus before or at birth [8]. One study conducted by the European Society of Pediatric and Neonatal Intensive Care COVID-19 Pediatric and Neonatal Registry (EPICENTRE) confirmed that the clinical characteristics of community-acquired cases and vertically-acquired cases in neonates differed [9]. The immune system continues to develop after birth and follows certain developmental patterns with age [10]. Because of the difference in the transmission mode of early-onset and late-onset infections and the continuous development of the immune system during the neonatal period, newborns with early-onset infection may respond differently to SARS-CoV-2 than those with late-onset infection. However, only a very limited number of studies have compared the symptoms, treatment, and prognosis of early-onset and late-onset SARS-CoV-2 infections in neonates, and differences in clinical characteristics of early-onset and late-onset SARS-CoV-2 infections in neonates remain unclear [11].

This study aimed to explore whether there are differences in the main clinical, radiological, and laboratory features of early-onset and late-onset neonatal SARS-CoV-2 infections to guide clinicians in the management of these neonates. We hypothesized that the clinical characteristics of early-onset and late-onset SARS-CoV-2 infections in neonates differed.

## Materials and methods

### Study participants

This prospective cohort study was performed at the Taizhou Hospital of Zhejiang Province in Linhai City, China. It began on December 7, 2022, when the Chinese government eased prevention and control measures enacted during the COVID-19 pandemic. We enrolled all neonates (*N* = 58) infected with SARS-CoV-2 within 28 days after birth and were hospitalized in the neonatal intensive care unit (NICU) of Taizhou Hospital from December 7, 2022, until January 3, 2023. The criteria for SARS-CoV-2 infection were positive real-time polymerase chain reaction results and/or antigen test results. According to the guideline issued by the Working Group for the Prevention and Control of Neonatal SARS-CoV-2 Infection in the Perinatal Period of the Editorial Committee of the Chinese Journal of Contemporary Pediatrics, neonates who exhibited symptoms with suspected SARS-CoV-2 infections were all admitted to the NICU at our center [12]. The exclusion criterion for this study was infection with pathogens other than SARS-CoV-2. SARS-CoV-2 infections were classified as early-onset (diagnosed within 7 days of birth) and late-onset (diagnosed more than 7 days after birth) cases.

### Data collection

We designed a data collection form with details regarding pregnancy, childbirth, signs and symptoms, laboratory test results, imaging results, treatments received by the neonate, and outcomes. This information was extracted from the electronic medical records and recorded by professional neonatologists.

Venous blood samples were collected for laboratory tests on the day of admission. Routine blood parameters, including the hemoglobin level, white blood cell count, neutrophil count (%), lymphocyte count (%), and platelet count, were detected using a routine analyzer (XN-350; SYSMEX, Hyogo, Kobe, Japan). C-reactive protein levels were measured using a BN ProSpec system (Siemens Healthcare Diagnostics, Erlangen, Germany). Biochemical parameters, including electrolytes, liver function, kidney function, and myocardial enzymes (except creatine kinase-myocardial band (MB)), were detected using an automatic biochemical analyzer (AU5821; Beckman Coulter, Brea, CA, USA). Creatine kinase-MB was detected using an automatic immunological analyzer (UniCel DXI-800; Beckman Coulter). Procalcitonin levels were determined using an electrochemiluminescence analyzer (Cobas e411; Roche, Basel, Switzerland).

According to the fifth edition of *Practical Neonatology*, the diagnosis of neonatal pneumonia is based upon a combination of clinical and radiographic [13]. Discharge criteria for neonates were as follows: all positive signs and symptoms lasted for more than 2 days and disappeared, and more than half of the lesions observed on chest radiography or chest CT were absorbed before discharge of patients with pneumonia.

This study was approved by the Ethics Committee of Taizhou Hospital of Zhejiang Province (approval number: k20230321). Data were collected after obtaining parental consent. All procedures were performed in accordance with the guidelines of our institutional ethics committee and complied with the tenets of the Declaration of Helsinki.

### Statistical analysis

Demographic characteristics were summarized using measures of central tendency, variability, and frequency. Appropriate statistical tests were applied to describe groups and compare the characteristics. Continuous variables with normal distribution were presented as the mean and standard deviation (SD) and compared using independent *t*-tests; otherwise, they were presented as the median and interquartile range (IQR) and compared using the Mann–Whitney U test. Categorical variables were presented as the frequency (percentage) and compared using the chi-square test.

The main outcomes (clinical symptoms, laboratory test results, treatments, and prognosis) were presented as the frequency (percentages) and compared using the chi-square test. The incidence of hospitalization attributable to SARS-CoV-2 infection was calculated using the denominator of the number of live births in Linhai from December 7, 2022, to January 3, 2023. No data were missing from this study. Statistical analyses were performed using IBM SPSS Statistics 23.0 (IBM Corp., Armonk, NY, USA). Statistical significance was set at *p* < 0.05 (two-sided).

## Results

### Incidence of COVID-19

Taizhou Hospital admitted all neonates in Linhai with SARS-CoV-2 infection who required hospitalization. From December 7, 2022, to January 3, 2023, 58 neonates infected with SARS-CoV-2 were hospitalized in the NICU of Taizhou Hospital. All 58 neonates roomed-in and were allowed to have skin-to-skin contact with their mothers at birth, but they were transferred to the NICU under strict respiratory and contact isolation without parental visitation when they exhibited clinical symptoms. During that period, the number of live births in Linhai was 546; therefore, the hospitalization incidence attributable to neonatal SARS-CoV-2 infection in Linhai was 10.6%. The first case was diagnosed on December 16, 2022. The number of diagnosed cases peaked at the end of December and began to decrease during early January.

### Demographic characteristics of hospitalized neonates infected with SARS-CoV-2

Among the 58 patients with SARS-CoV-2 diagnosis, the median age at diagnosis was 18 days (IQR: 6.75–25.25 days). Sixteen (28%) were early-onset cases, and 42 (72%) were late-onset cases. The youngest infected neonate was 2 days old. Of the 58 patients with SARS-CoV-2 diagnosis, 35 (60%) were male patients. The gestational age at birth was 268 ± 10 (mean ± SD) days. Most patients (67%; 39 of 58) were born via vaginal delivery; the others (33%; 19 of 58) were born via cesarean delivery. Most patients (62%; 36 of 58) were fed by human milk. The birth weight of all neonates included in this study ranged from 1900 to 4580 g (mean ± SD: 3163 ± 488 g). The median age of the mothers of the enrolled neonates was 29 years (IQR: 26–32 years). Among the 58 mothers of the patients, 18 (31%) received three doses of the COVID-19 vaccine, 25 (43%) received two doses of the COVID-19 vaccine, 8 (14%) did not undergo vaccination, and 7 (12%) received one dose of the COVID-19 vaccine. This information did not differ between the early-onset and late-onset groups (Table 1).

**Table 1:** Differences in demographic characteristics of neonates with early-onset and late-onset SARS-CoV-2 infections (*N* = 58)

**Demographic characteristics**	**Total (*N* = 58)**	**Early-onset (*N* = 16)**	**Late-onset (*N* = 42)**	***p*-value**
**Neonatal factors**				
**Gestational age at birth, days, mean ± SD**	268 ± 10	269 ± 6	268 ± 11	0.815
**Sex, *N* (%)**				
Male	35 (60)	12 (75)	23 (55)	0.232
Female	23 (40)	4 (25)	19 (45)	
**Delivery mode, *N* (%)**				
Vaginal delivery	39 (67)	8 (50)	31 (74)	0.119
Cesarean delivery	19 (33)	8 (50)	11 (26)	
**Feeding pattern, *N* (%)**				
Breastfeeding	36 (62)	11 (69)	25 (60)	0.517
Formula feeding	22 (38)	5 (31)	17 (40)	
**Birth weight, mean ± SD**	3163 ± 488	3139 ± 417	3172 ± 517	0.822
**Days of hospitalization, median (IQR)**	6 (4.75–7.00)	6 (4.26–7.00)	5.5 (4.75–7.00)	0.664
**Maternal factors**				
**Maternal age, years, median (IQR)**	29 (26–32)	29 (28–34)	29 (25–31)	0.225
**SARS-CoV-2 vaccine status**^**a**^, ***N* (%)**				
Unvaccinated	8 (14)	5 (31)	3 (7)	0.128
1 dose	7 (12)	2 (13)	5 (12)	
2 doses	25 (43)	5 (31)	20 (48)	
3 doses	18 (31)	4 (25)	14 (33)	
**Maternal COVID status, *N* (%)**				
SARS-CoV-2 positive mothers at birth	11 (19)	10 (63)	1 (2)	< 0.001
SARS-CoV-2 positive mothers at the time of NICU hospitalization	45 (78)	14 (88)	31 (74)	0.318

*IQR* interquartile range, *SARS-CoV-2* severe acute respiratory syndrome coronavirus 2, *SD* standard deviation, *COVID* coronavirus disease

^a^The vaccinations given to the mothers were Sinovac-CoronaVac COVID-19 vaccine recognized by the World Health Organization

The proportion of mothers testing positive for SARS-CoV-2 at delivery in the early-onset group (63%; 10 of 16) was significantly more than that in the late-onset group (2%; 1 of 4; *p* < 0.001). The proportion of mothers testing positive for SARS-CoV-2 at the time of NICU hospitalization did not differ between the early-onset (88%; 14 of 16) and late-onset groups (74%; 31 of 42; *p* = 0.318). Table 1 detailed information on maternal COVID status.

### Differences in symptoms and treatments of neonates with early-onset and late-onset SARS-CoV-2 infections

Neonates with early-onset infection experienced different symptoms than those with late-onset infection (Table 2). The most common symptom among neonates with early-onset infection was jaundice, which appeared in all neonates (100%; 16 of 16) with early-onset infection but in only 10% (4 of 42) of neonates with late-onset infection (*p* < 0.001). In the late-onset group, the most common symptoms were fever and cough, with incidences of 91% (38 of 42) and 62% (25 of 42), respectively. However, the incidences of fever and cough among neonates with early-onset infection were only 44% (7 of 16; *p* < 0.001) and 6% (1 of 16; *p* < 0.001), respectively. In addition to these symptoms, coryza (6%; 1 of 16) and apnea (6%; 1 of 16) were observed in the early-onset group, and coryza (29%; 12 of 42), poor feeding (21%; 9 of 42), tachypnea (14%; 6 of 42), apnea (2%; 1 of 42), and vomiting (2%; 1 of 42) were observed in the late-onset group. However, the incidences of these symptoms were not statistically different between groups.

**Table 2:** Differences in symptoms of neonates with early-onset and late-onset SARS-CoV-2 infections (*N* = 58)

**Symptoms**	**Early-onset **(*N* = 16)			**Late-onset **(*N* = 42)			***p*-value**
	**Cases (no.)**	**Clinical symptom proportion (%)**	**Clinical symptom incidence (%)**	**Cases (no.)**	**Clinical symptom proportion (%)**	**Clinical symptom incidence (%)**	
Total clinical symptoms	26	100	–	96	100	–	–
Jaundice^a^	16	62	100	4	4	10	< 0.001
Fever	7	27	44	38	40	91	< 0.001
Cough	1	4	6	25	26	62	< 0.001
Coryza	1	4	6	12	13	29	0.087
Poor feeding	0	0	0	9	9	21	0.051
Tachypnea	0	0	0	6	6	14	0.173
Apnea	1	4	6	1	1	2	0.479
Vomiting	0	0	0	1	1	2	1.000

*SARS-CoV-2* severe acute respiratory syndrome coronavirus 2

^a^Considering that physiological jaundice peaks around 48–72 h from birth, we compared both groups with non-COVID NICU population at a matched age during the same period of time and found that there was no statistically significant difference; therefore, we believed that the different incidence of jaundice between the two groups was due to the difference of age

Our study confirmed that there was no difference in the treatment administered to the two groups during hospitalization; however, the clinical symptoms differed (Table 3). Most neonates (early-onset group, 75% (12 of 16); late-onset group, 60% (25 of 42)) did not receive treatment during hospitalization. Neonates in the early-onset group (19%; 3 of 16) and those in the late-onset group (36%; 15 of 42) received antibiotic infusion during hospitalization. A small percentage of neonates in these two groups received respiratory support (early-onset group, 6% (1 of 16); late-onset group, 5% (2 of 42)), corticosteroids (early-onset group, 6% (1 of 16); late-onset group, 5% (2 of 42)), immunoglobulin infusion (early-onset group, 6% (1 of 16); late-onset group, 0% (0 of 42)), blood transfusions (early-onset group, 6% (1 of 16); late-onset group, 0% (0 of 42)), and liver protected-drugs (early-onset group, 0% (0 of 16); late-onset group, 5% (2 of 42)).

**Table 3:** Differences in treatments received between neonates in the early- and late-onset SARS-CoV-2 infection groups (*N* = 58)

**Treatments**	**Early-onset **(*N* = 16)			**Late-onset **(*N* = 42)			***p*-value**
	**Cases (no.)**	**Proportion of treatments (%)**	**Incidence of treatments (%)**	**Cases (no.)**	**Proportion of treatments (%)**	**Incidence of treatments (%)**	
Total treatments	19	100	–	46	100	–	–
Antibiotics	3	16	19	15	33	36	0.342
Respiratory support	1	5	6	2	4	5	1.000
Corticosteroids	1	5	6	2	4	5	1.000
Immunoglobulin	1	5	6	0	0	0	0.276
Blood transfusions	1	5	6	0	0	0	0.276
Liver protected-drugs	0	0	0	2	4	5	1.000
No special treatment	12	63	75	25	54	60	0.365

*SARS-CoV-2* severe acute respiratory syndrome coronavirus 2

### Differences in hematological and biochemical results of neonates with early-onset and late-onset SARS-CoV-2 infections

This study compared laboratory results of the early-onset and late-onset groups. The levels of hemoglobin (early-onset group, mean ± SD: 180 ± 18; late-onset group, mean ± SD: 139 ± 19; *p* < 0.001), white bold cell count (early-onset group, mean ± SD: 9.2 ± 2.9; late-onset group, mean ± SD: 7.2 ± 2.7; *p* = 0.019), neutrophil count (early-onset group, mean ± SD: 4.8 ± 2.0; late-onset group, mean ± SD: 2.8 ± 1.5; *p* < 0.001), procalcitonin (early-onset group, mean ± SD: 0.28 ± 0.21; late-onset group, mean ± SD: 0.15 ± 0.09; *p* = 0.039), alanine aminotransferase (early-onset group, mean ± SD: 15 ± 9; late-onset group, mean ± SD: 23 ± 10; *p* = 0.009), total bilirubin (early-onset group, mean ± SD: 253.2 ± 69.7; late-onset group, mean ± SD: 119.6 ± 74.8; *p* < 0.001), creatinine (early-onset group, mean ± SD: 42 ± 9; late-onset group, mean ± SD: 27 ± 5; *p* < 0.001), urea (early-onset group, mean ± SD: 2.22 ± 1.22; late-onset group, mean ± SD: 3.48 ± 1.31; *p* = 0.002), creatine kinase-MB (early-onset group, mean ± SD: 10.83 ± 4.60; late-onset group, mean ± SD: 5.73 ± 2.00; *p* < 0.001), and lactate dehydrogenase (early-onset group, mean ± SD: 696 ± 255; late-onset group, mean ± SD: 377 ± 128; *p* < 0.001) were significantly different between the two groups. The remaining parameters were not significantly different between the early-onset and late-onset groups (Table 4).

**Table 4:** Differences in hematological and biochemical results of neonates with early-onset and late-onset SARS-CoV-2 infections

**Parameter**	**Early-onset** (*N* = 16)	**Late-onset** (*N* = 42)	***p*-value**
	**Mean ± SD**	**Mean ± SD**	
Hemoglobin (g/L)	180 ± 18	139 ± 19	< 0.001
White blood cell count (10^9^/L)	9.2 ± 2.9	7.2 ± 2.7	0.019
Neutrophil count (10^9^/L)	4.8 ± 2.0	2.8 ± 1.5	< 0.001
Lymphocyte count (10^9^/L)	2.9 ± 1.6	2.9 ± 1.8	0.964
Platelet count (10^9^/L)	258 ± 94	286 ± 7.9	0.270
C-reactive protein (mg/L)	2.8 ± 2.2	1.9 ± 2.3	0.207
Procalcitonin (ng/mL)	0.28 ± 0.21	0.15 ± 0.09	0.039
Alanine aminotransferase (U/L)	15 ± 9	23 ± 10	0.009
Aspartate aminotransferase (U/L)	68 ± 34	50 ± 22	0.071
Total bilirubin (umol/L)	253.2 ± 69.7	119.6 ± 74.8	< 0.001
Creatine kinase-MB (ng/mL)	10.83 ± 4.60	5.73 ± 2.00	< 0.001
Lactate dehydrogenase (U/L)	696 ± 255	377 ± 128	< 0.001
Creatinine (umol/L)	42 ± 9	27 ± 5	< 0.001
Urea (mmol/L)	2.22 ± 1.22	3.48 ± 1.31	0.002

*MB* myocardial band, *SARS-CoV-2* severe acute respiratory syndrome coronavirus 2, *SD* standard deviation

### Differences in outcomes of the early-onset and late-onset groups

**Fig. 1:**
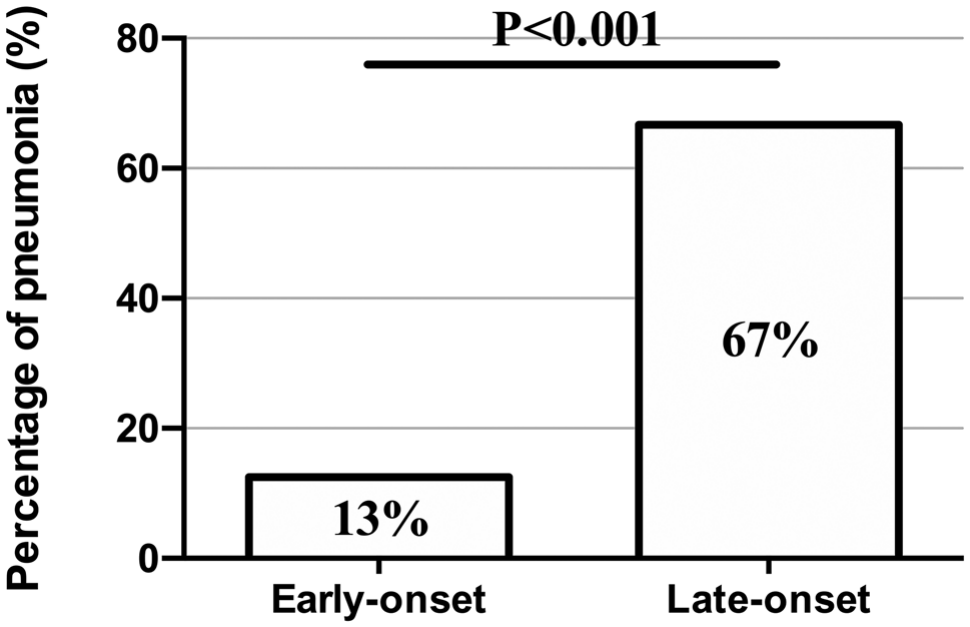
Neonates with late-onset severe acute respiratory syndrome coronavirus 2 (SARS-CoV-2) infection are more likely to develop pneumonia (*N *= 58)

Our study confirmed that the neonatal SARS-CoV-2 infection prognosis was good. All 58 patients enrolled in this study recovered and were discharged from the hospital. However, neonates with late-onset SARS-CoV-2 infection were more likely to develop pneumonia (early-onset group: 13% (2 of 16); late-onset group: 67% (28 of 42); *p* < 0.001) (Fig. 1). There was no significant difference (*p* = 0.664) in the hospitalization lengths of the groups (Table 1). The median length of stay for neonates in the early-onset group was 6 days (IQR: 4.26–7.00 days), and that for the late-onset group was 5.5 days (IQR: 4.75–7.00 days).

## Discussion

This study clarified the prevalence of neonatal SARS-CoV-2 infection in Linhai within 1 month of the Chinese government easing the prevention and control measures enacted during the COVID-19 pandemic. The SARS-CoV-2 variant that caused this pandemic in China was omicron, and the incidence of hospitalization for neonatal SARS-CoV-2 infection in Linhai was 10.6%. A systematic review including 7480 children with SARS-CoV-2 infection from January 1, 2020, to May 1, 2020, found that only 25 patients were neonates [14]. A prospective population-based cohort study performed in the UK confirmed that inpatient care for neonates with confirmed SARS-CoV-2 infection was rare, with 5.6 cases per 10,000 live births in March and April 2020 [5]. Our study showed a higher incidence of neonatal SARS-CoV-2 infection in Linhai during the end of the COVID-19 pandemic than during the beginning of the COVID-19 pandemic.

Guidelines for managing neonates born to SARS-CoV-2 positive mothers during pandemics were very different between countries [15, 16]. Our hospital managed neonates during the COVID-19 epidemic according to the guideline issued by the Working Group for the Prevention and Control of Neonatal SARS-CoV-2 Infection in the Perinatal Period of the Editorial Committee of Chinese Journal of Contemporary Pediatrics [12]. In line with other countries, SARS-CoV-2-positive women were admitted to a designated labor room or operating theater, and N95 face masks, goggles, gowns, and gloves were necessary for newborn resuscitation in our hospital [16]. Like Japan and South Africa, only if neonates were symptomatic, we performed virologic testing on them [16]. Unlike most countries, neonates in our hospital born to SARS-CoV-2 positive mothers were separated from their mothers and isolated in the NICU only if they exhibited symptoms of suspected SARS-CoV-2 infection, and the rest of them were roomed-in and were allowed to have skin-to-skin contact with their mothers [16]. In our hospital, neonates were isolated in a single room after admission to the NICU, which was the same as in most countries [15]. Like in Poland, all family members did not have access to the NICU, and staff would send photos of neonates by text when neonates were transferred to the NICU in our hospital [15]. Except for South Korea and Singapore, breastfeeding of neonates with breastmilk from asymptomatic but infected mothers was encouraged in most countries, which was encouraged in our country as well [16]. Management of newborns during the COVID-19 pandemic varies from country to country, so researches are still needed to establish guidelines with high-level evidence.

Family-centered care practices such as breastfeeding, co-habitation, and skin-to-skin contact are critical to the well-being of neonates, but the COVID-19 pandemic had a negative impact on family-centered care [17]. All 58 neonates in this study were roomed-in and were allowed to have skin-to-skin contact with their mothers at birth, but they were transferred to the NICU under strict respiratory and contact isolation without parental visitation when they exhibited clinical symptoms. Therefore, the COVID-19 pandemic had a negative impact on family-centered care in patients enrolled in this study.

Previous study confirmed that placental inflammation occurred in cases of transplacental transmission, which may be associated with perinatal asphyxia and an increased rate of cesarean delivery [18]. But there was no difference in the incidence of cesarean section between the two groups, and perinatal asphyxia was not observed in this study, which may be related to the absence of cases with transplacental infection in the early-onset group.

The results of our study suggest that neonates with early-onset SARS-CoV-2 infection present with different symptoms than neonates with late-onset infection. Fever and cough were more common in late-onset cases than early-onset cases. In line with the findings of our study, a cohort study conducted by the EPICENTRE group concluded that fever and cough were more common in community-acquired patients than vertically-acquired patients [9]. But rhinitis and feeding difficulties were more common in community-acquired cases than vertically-acquired patients too, which was different from ours [9]. In addition to the above symptoms, the patients also reported lethargy, tachycardia, hemodynamic instability, cardiomyopathy, hypothermia, and intra-ventricular hemorrhage, which we did not observe in our study [9]. Like our findings, a meta-analysis of neonatal COVID reported that neonates infected with SARS-CoV-2 developed fever, gastrointestinal symptoms, and respiratory symptoms [19]. But they reported neurological manifestations, which were not observed in our study [19]. The only previous study comparing the clinical characteristics of neonates with vertically-acquired and community-acquired SARS-CoV-2 infection was the study conducted by the EPICENTRE group, so further research is needed to clarify the differences in clinical symptoms between the two [9].

Based on statistical data, pneumonia is more common in neonates with early-onset SARS-CoV-2 infection than in those with late-onset infection. Few studies have reported early-onset or late-onset infections in neonates. Only three cases of early-onset SARS-CoV-2 infection in neonates presenting with pneumonia have been reported [8]. Therefore, further studies are necessary to clarify the differences in the incidence of pneumonia between the early-onset and late-onset groups.

Most patients included in our study did not receive treatment during their stay in our NICU, and the main reason for their admission to the NICU was to allow isolation, observation, and monitoring. Some patients were on antibiotics because of probable or confirmed bacterial infection according to the study by De Rose et al. [20]. At our center, we assessed the probable or confirmed bacterial infection based on the fifth edition of *Practical Neonatology* [13]. Patients with 2 or more positive nonspecific blood tests (C-reactive protein > 8 mg/L, procalcitonin > 1.0 ng/mL, or white blood cell count ≥ 20×10^9^/L) were considered to have possible bacterial infection, and patients tested positive for sterile sample (neonatal blood, lower respiratory tract samples obtained by tracheal aspirate, or cerebrospinal fluid) cultures were considered to have confirmed bacterial infection. One patient was treated with immunoglobulins because of hemolytic jaundice. Inhaled corticosteroids were used to relieve airway inflammation in three patients. All neonates were discharged after a median hospital stay of 6 days. Our study suggested that there was no difference in the treatment administered to the two groups during hospitalization. In contrast to our study, the EPICENTRE group found that community-acquired cases received antibiotics more frequently than vertically-acquired patients [9]. Current research on the differences in treatments between neonates with early-onset and late-onset SARS-CoV-2 infections is quite limited, so further research is needed to clarify the differences between the two.

One cohort study found that human milk from lactating mothers with SARS-CoV-2 infection or SARS-CoV-2 vaccination contributed to neutralization activity against SARS-CoV-2 [21]. Conti et al. found that mothers infected with SARS-CoV-2 in the peripartum period could protect the newborn via breastmilk secretory immunoglobulin A (IgA) [22]. Both of these studies suggested that it would be clinically beneficial for newborns to receive human milk from mothers with COVID-19 infection or who are vaccinated. All 58 patients enrolled in this study recovered, and there were no severe cases, which may also be related to the fact that most of the patients were fed by human milk.

The mechanisms of vertically- and community-acquired infections are different. EPICENTRE group confirmed that the clinical characteristics of community-acquired cases and vertically-acquired cases in neonates differed [9]. Vertical transmission of SARS-CoV-2 includes “in-utero” transmission and “intrapartum” transmission [11]. “In-utero” transmission of SARS-CoV-2 occurs when transmembrane protein angiotensin-converting enzyme 2 (ACE2) and the transmembrane protease serine 2 (TMPRSS2) are expressed on cellular membranes in placental and fetal tissues [11, 20]. Previous study suggested that the placenta at term expressed ACE2 moderately but not TMPRSS2, so they believed that transplacental transmission rarely occurred at term but raised concern about preterm infection [23]. Piersigilli et al. found a 26-week preterm neonate occurred horizontal SARS-CoV-2 infection under standard NICU precautions [24]. Therefore, both vertical and horizontal transmission should be emphasized in preterm infants.

Differences in clinical characteristics of neonates with early-onset SARS-CoV-2 infection and those with late-onset SARS-CoV-2 infection are considered related to dynamic changes in maternal SARS‐CoV‐2 immunoglobulin G (IgG) acquired in neonates. It has been shown that IgG produced by maternal SARS-CoV-2 infection or vaccination can be transferred to the fetus via the placenta [25, 26]. A previous study suggested that maternal SARS-CoV-2 IgG levels in neonates decreased sharply after birth [27]. Therefore, we believe that the lower risk of pneumonia and milder symptoms for neonates with early-onset SARS-CoV-2 infection compared to those for neonates with late-onset SARS-CoV-2 infection were attributable to the greater protective effects of maternal SARS-CoV-2 IgG in neonates with early-onset SARS-CoV-2 infections.

Based on the study[9] of the EPICENTRE group, this study deepens our understanding of early-onset and late-onset SARS-CoV-2 infections in neonates. This study had some limitations. First, this was a single-center study which was weaker than the EPICENTRE multi-center study design, so the sample was not representative, and it is very difficult to generalize the results [28]. Therefore, additional multicenter studies are necessary. Second, all clinical characteristics presented in this study are those of patients who sought medical treatment, thus leading to selection bias. Third, positive results of antigen tests, which have poor specificity and may result in false-positive results, were an inclusion criterion. Fourth, the basic characteristics of the two groups were similar, so we did not perform a multivariate analysis, but some confounders such as mothers’ perinatal medication that we were unable to collect may affect our results. Fifth, we did not perform serology (IgA, immunoglobulin M (IgM), and IgG), and the sample used for virologic testing was not sterile and was only collected once when the patient was symptomatic. We were unable to categorize the cases into transplacental, vaginal, and post-natal infection based on WHO criteria, and misclassification may have occurred [29].

In conclusion, the present study confirmed that the clinical characteristics of early-onset and late-onset SARS-CoV-2 infections in neonates differed. Fever and cough were the most common symptoms among neonates with late-onset infection. Neonates with late-onset SARS-CoV-2 infection are more likely to develop pneumonia. Different clinical management is necessary for neonates with early-onset and late-onset SARS-CoV-2 infections. The long-term associations between early-onset and late-onset neonatal SARS-CoV-2 infections and the lifespan, health, and well-being of children should be investigated further.

## Data Availability

The data supporting the findings of this study are available from the corresponding author upon reasonable request.

## Abbreviations

ACE: Transmembrane proteins angiotensin-converting enzyme 2
COVID-19: Coronavirus disease
CT: Computed tomography
EPICENTRE: European Society of Pediatric and Neonatal Intensive Care COVID-19 Pediatric and Neonatal Registry
IgA: Immunoglobulin A
IgM: Immunoglobulin M
IgG: Immunoglobulin G
IQR: Interquartile range
MB: Myocardial band
NICU: Neonatal intensive care unit
SARS-CoV-2: Severe acute respiratory syndrome coronavirus 2
SD: Standard deviation
TMPRSS2: The transmembrane protease serine 2
WHO: World Health Organization
